# Nanostructured Al-ZnO/CdSe/Cu_2_O ETA solar cells on Al-ZnO film/quartz glass templates

**DOI:** 10.1186/1556-276X-6-614

**Published:** 2011-12-04

**Authors:** Xianghu Wang, Rongbin Li, Donghua Fan

**Affiliations:** 1 School of Mechanical Engineering, Shanghai Dianji University, 1201 Jiang Chuan Road, Shanghai, 200245, People's Republic of China; 2 Institute of Functional Materials Research and Department of Mathematics and Physics, Wuyi University, Jiangmen, 529020, People's Republic of China

**Keywords:** extremely thin absorber, oxide semiconducting material, window material for solar cell

## Abstract

The quartz/Al-ZnO film/nanostructured Al-ZnO/CdSe/Cu_2_O extremely thin absorber solar cell has been successfully realized. The Al-doped ZnO one-dimensional nanostructures on quartz templates covered by a sputtering Al-doped ZnO film was used as the n-type electrode. A 19- to 35-nm-thin layer of CdSe absorber was deposited by radio frequency magnetron sputtering, coating the ZnO nanostructures. The voids between the Al-ZnO/CdSe nanostructures were filled with p-type Cu_2_O, and therefore, the entire assembly formed a p-i-n junction. The cell shows the energy conversion efficiency as high as 3.16%, which is an interesting option for developing new solar cell devices.

**PACS**: 88.40.jp; 73.40.Lq; 73.50.Pz.

## Introduction

Extremely thin absorber [ETA] solar cells have attracted much attention because of their probability to be low-cost solar cells. It consists of a nano- or microstructured layer which also serves as an n-type window layer to the cell, an absorber (1.1 <*E*_g _< 1.8 eV) that is conformably deposited on the layer, and a void-filling p-type material with a metallic back contact. Oxide semiconducting materials have potential applications in ETA solar cells as p- and n-type widow layers due to their many excellent physical properties and economic values, such as having a wide bandgap, good thermal stability, and being a low-cost and environment-friendly material. However, it is difficult to achieve p- and n-type oxide semiconductors simultaneously. One-dimensional [1-D] nanostructured zinc oxide [ZnO] semiconducting materials, which are intrinsic n-type wide-bandgap semiconductors with a direct bandgap energy of 3.37 eV and exciton bounding energy of 60 meV [1], are specially suitable for n-type electrode materials of solar cells [2-8]. When 1-D ZnO is used as an n-type electrode material for solar cells, it has a number of advantages, such as a high transmittance in the visible wavelength region, a larger surface area, a high electron mobility along the growth direction, and a highly efficient electron transport [9]. However, the p-type ZnO electrode material has to be replaced by other semiconducting materials due to the difficulty in the growth of p-type ZnO. ETA solar cells with a ZnO nanostructure as n-type window material have reached the efficiency of 2.3% to 3.4% [2-4], but the p-type window material is CuSCN in these reported n-ZnO/absorber/p-CuSCN-structured ETA solar cells. In the process of such ETA solar cell fabrication, CuSCN is in the upper part of the absorber layer. When the fabrication of the absorber layer was finished, the synthesis of CuSCN encountered unexpected problems, such as homogeneity and reproducibility problems, during solution deposition. Cuprous oxide [Cu_2_O] is a natural p-type direct gap semiconductor with a bandgap energy of 2.1 eV [6]. It has been predicated that Cu_2_O is promising for photovoltaic applications, with a theoretical energy conversion efficiency of 20% [10]. Many papers have reported the fabrication of solar cells used in ZnO and Cu_2_O semiconducting materials as n- and p-type window layers [11-13], but the energy conversion efficiency is less than 1.5%. The realizations of such solar cells favor the fact that Cu_2_O is potentially useful for the application in the p-type layer of ETA solar cells.

In this study, we report the preparation and characterization of nanostructured Al-ZnO/CdSe/Cu_2_O solar cells on a Al-ZnO film/quartz glass template with the energy conversion efficiency as high as 3.16%.

## Materials and methods

**Figure 1:**
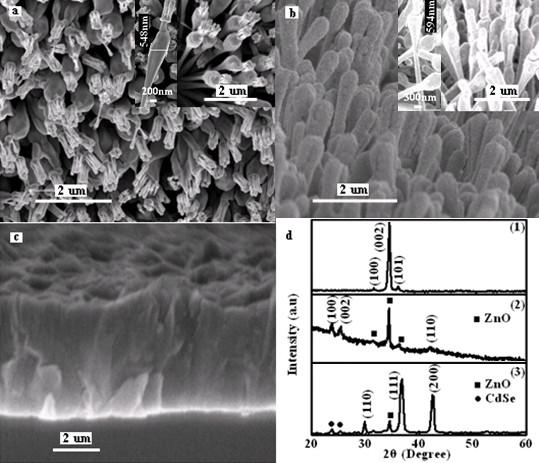
**SEM images**. (**a**) Top view of quartz/Al-ZnO film/Al-ZnO nanostructures, (**b**) top view of Al-ZnO nanostructures covered by CdSe, (**c**) cross-sectional view of Cu_2_O covering the Al-ZnO nanostructures/CdSe, and (**d**) XRD patterns of (1) quartz/Al-ZnO film/Al-ZnO nanostructures, (2) quartz/Al-ZnO film/Al-ZnO nanostructures/CdSe, and (3) quartz/Al-ZnO film/Al-ZnO nanostructures/CdSe/Cu_2_O.

The well-aligned, single crystalline, vertical, and 1-D Al-doped ZnO nanostructures [Al-ZnO] used in this study were grown on quartz templates/Al-ZnO film. Detailed growth procedures could be found elsewhere [14]. It should be noted that the sputtered Al-ZnO film is electrically conductive and can serve as the n-contact layer with a Hall mobility of 18.86 cm^2 ^V^-1 ^s^-1 ^and a carrier concentration of 7.34 × 10^19 ^cm^-3^. The CdSe layer was subsequently deposited on the surface of the quartz templates/Al-ZnO film/Al-doped ZnO nanostructures by radio frequency [RF] magnetron sputtering. Pure CdSe of 99.999% was used as the target. The sputtering gas is high pure Ar, and the substrate temperature is 500 K. The growth chamber was pumped down to a base pressure of about 2 × 10^-4 ^Pa initially and was then filled with Ar up to 0.8 Pa. The sputtering power and time are 18 W and 3 min, respectively. The CdSe layer with a thickness in the range of 19 to 35 nm has been estimated from a statistical evaluation of the Al-doped ZnO nanostructures before and after CdSe deposition, as shown in Figure 1. Cu_2_O film was subsequently deposited on top of the quartz/Al-ZnO film/nanostructured Al-ZnO/CdSe by RF magnetron sputtering. Pure Cu of 99.999% was used as the target. The sputtering gas is the mixed Ar/O_2 _with a flow rate of 20/13, and the substrate temperature is 580 K. The sputtering power and time are 16 W and 1.5 h, respectively.

The morphology and structure of the as-synthesized samples were characterized using field-emission scanning electron microscopy (NoVaTM Nano SEM 430, Shanghai NanoVis Electronics Technologies Ltd., Co., Shanghai, China) and X-ray diffraction [XRD] (X'Pert Pro with CuKα1 of 1.5406 Å; X'Pert, PANalytical B.V., Almelo, The Netherlands). Optical transmission was carried out using a UV-3101PC spectrometer (Shimadzu Corporation, Nakagyo-ku, Kyoto, Japan). Photo conversion was measured using the Newport model 69907; the light source was a xenon lamp with a 100-mW/cm^2 ^power at an air mass [AM] of 1.5. The light illuminated the samples from the glass substrate side with a contact area of 0.71 cm × 0.52 cm to 0.369 cm^2^. Electrodes were fabricated by depositing metal indium on the Al-ZnO film/quartz templates and on the gold/nickel on Cu_2_O film by conventional vacuum evaporation and sintering in vacuum (≤ 10^-5 ^Torr).

## Results and discussion

Figure 1a shows the morphologies of the quartz/Al-ZnO film/Al-ZnO nanostructures, indicating that the highly densified Al-ZnO hierarchical nanostructures are quasi-perpendicular to the substrate, and the typical density of the nanostructures is approximately 6 × 10^9 ^cm^-2^. The Al-ZnO nanostructures comprised three segments, namely a long single nanorod at the bottom, a thin cap in between, and several nanorods on top, respectively. The single nanorod is about 3 μm in length. The diameter of the nanorod is uneven and becomes larger and larger near the thin cap. The thin cap is about 50 nm in thickness with a diameter of approximately 400 nm. The nanorods on top have a hexagonal morphology with a length of approximately 600 nm. An absorber layer of CdSe was evenly deposited on the surface of the quartz/Al-ZnO film/Al-ZnO nanostructure by RF magnetron sputtering. The thickness of CdSe was estimated as half the difference value between the diameter of CdSe-coating ZnO nanostructure in the largest part and the diameter of the pristine ZnO nanostructure. The thickness of the CdSe layer was estimated in the range of 19 to 35 nm based on multimetering. The diameter of a single ZnO nanostructure was measured by the size label system of NoVaTM Nano SEM 430 (Shanghai NanoVis Electronics Technologies Ltd., Co., Shanghai, China), as shown in the insets of Figure 1a, b. The Cu_2_O film was subsequently deposited on the surface of the quartz/Al-ZnO film/nanostructured Al-ZnO/CdSe/by RF magnetron sputtering; the cross-sectional view shows that the sample is compact and dense, as shown in Figure 1c.

The XRD patterns are shown in Figure 1d. Figure 1d(1) is the XRD pattern of the quartz/Al-ZnO film/Al-ZnO nanostructures. All the peaks can be indexed to the hexagonal wurtzite structure of ZnO, and no other detectable phases exist in the Al-ZnO hierarchical nanostructures. After depositing the CdSe layer on the surface of the quartz/Al-ZnO film/nanostructured Al-ZnO, the characteristic (100), (002), and (110) peaks of CdSe were detected beside the peaks of the ZnO in Figure 1d(2), showing that the CdSe layer constituted single hexagonal phases. Figure 1d(3) shows the results of the quartz/Al-ZnO film/nanostructured Al-ZnO/CdSe/Cu_2_O. It can be found that the characteristic (110), (111), and (200) peaks belong to Cu_2_O with a cubic structure beside the peaks of ZnO and CdSe.

**Figure 2:**
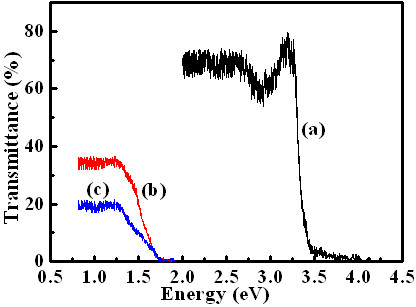
**The optical transmittance spectra**. (**a**) Quartz/Al-ZnO film/Al-ZnO nanostructures, (**b**) quartz/Al-ZnO film/Al-ZnO nanostructures/CdSe, and (**c**) quartz/Al-ZnO film/Al-ZnO nanostructures/CdSe/Cu_2_O.

Figure 2 shows the optical transmittance of the Al-ZnO hierarchical nanostructures on the quartz substrate with ZnO film. The transmittance is above 60% in the visible region. After depositing on the CdSe and Cu_2_O layer, the transmittance was decreased to 19%. Energy gaps (*E*_g_) close to 3.372 eV for the Al-ZnO hierarchical nanostructures and 1.67 eV for CdSe have been estimated from these spectra using the relation for direct transition [15]. The optical bandgap of the Cu_2_O layer in the cell could not have been measured because it was deposited on the CdSe layer, but it has a bandgap of 2.1 eV [16], which is larger than that of the CdSe.

**Figure 3:**
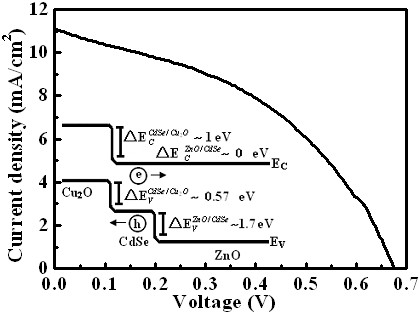
***J-V *curves**. The Al-ZnO nanostructures/CdSe/Cu_2_O solar cell *J*-*V *curves under illumination with a 100-mW/cm^2 ^power at 1.5 AM. Inset is the relative band location diagram of nanostructure Al-ZnO/CdSe/Cu_2_O heterostructure. *E_C _*and *E_V _*are the conduction and valence bands, respectively.

The photovoltaic properties of the quartz/Al-ZnO film/nanostructured Al-ZnO/CdSe/Cu_2_O solar cell were measured under illumination with a 100-mW/cm^2 ^power at 1.5 AM from the contact with area of 0.369 cm^2^. Figure 3 shows the current density-voltage [*J*-*V*] measurements for the solar cell. Prior to the measurement for the photovoltaic properties, the *J*-*V *curve measured between the front contact of the evaporated indium on the Al-ZnO film and the back contact of the evaporated gold/nickel on Cu_2_O was rectifying. The junctions (ZnO/CdSe, ZnO/Cu_2_O, CdSe/Cu_2_O) were also rectifying. The short-circuit photocurrent density [*J*_SC_], open circuit voltage [*V*_OC_], fill factor [FF], and conversion efficiency of the fabricated solar cell were 11.09 mA cm^-2^, 0.67 V, 0.45, and 3.16%, respectively. These values of *J*_SC_, *V*_OC_, FF, and efficiency are the highest to be reported for the oxide used as p- and n-type window layers simultaneously in ETA solar cells. The relative band location diagram of the nanostructured Al-ZnO/CdSe/Cu_2_O heterostructure is shown in the inset of Figure 3, using the reported values for band structure relationship between ZnO, CdSe, [7] and Cu_2_O [6]. According to the band location diagram, it was found that the valence band offsets at the interfaces of ZnO/CdSe(${\Delta E}_{V}^{\text{ZnO/CdSe}}$), CdSe/Cu_2_O(${\Delta E}_{V}^{\text{CdSe/C}{\text{u}}_{\text{2}}\text{O}}$), and ZnO/Cu_2_O(${\Delta E}_{V}^{\text{ZnO/C}{\text{u}}_{\text{2}}\text{O}}$) heterostructures were around 1.7, 0.57, and 2.27 eV; the conduction band offsets at the interfaces of ZnO/CdSe(${\Delta E}_{C}^{\text{ZnO/CdSe}}$), CdSe/Cu_2_O(${\Delta E}_{C}^{\text{CdSe/C}{\text{u}}_{\text{2}}\text{O}}$), and ZnO/Cu_2_O(${\Delta E}_{C}^{\text{ZnO/C}{\text{u}}_{\text{2}}\text{O}}$) heterostructures were around 0, 1, and 1 eV, respectively. Due to the short diffusion length of the p-type Cu_2_O and n-type ZnO semiconductors employed in the p-i-n structure, a CdSe absorption layer is introduced between the p- and n-type layers, inducing an internal electric field across the absorption layer, which promotes electron transfer from the CdSe absorber to the ZnO and hole transfer to Cu_2_O.

**Figure 4:**
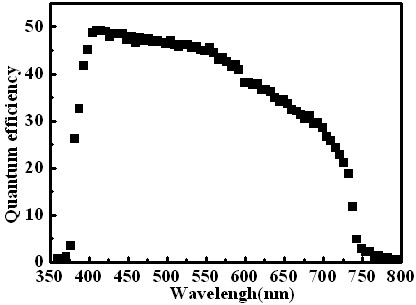
**The spectral response of the solar cell**.

Figure 4 shows the spectral response of the solar cell. The quantum efficiency was measured in the wavelength range from 382 to 730 nm. The wavelength range where the cells show the quantum efficiency is higher than the energy gap of the CdSe, indicating that the photons are mainly absorbed in the CdSe layer. The largest value of quantum efficiency is close to 49% at wavelengths of about 400 nm. At the wavelength of 590 nm, the value obviously decreases to 38%. Above 730 nm, the quantum efficiency of the cell is very low. It will be presumed that beside the most part of photons absorbed in the layer of CdSe, in the region with high quantum efficiency (between 400 nm and 590 nm), a part of the photons are also absorbed in the depletion layer region of the heterojunction between the nanostructured Al-ZnO/CdSe where the high electric field causes a high collection probability. In the region between 590 nm and 730 nm, a part of the photons are also absorbed in the depletion layer region of the heterojunction between CdSe/Cu_2_O.

## Conclusions

To summarize, the quartz/Al-ZnO film/nanostructured Al-ZnO/CdSe/Cu_2_O solar cell was fabricated. The oxide semiconducting materials of the ZnO nanostructure and Cu_2_O were simultaneously used as n- and p-type window layers; CdSe was used as absorber layer. Although the transmittance of the Al-ZnO nanostructure is only about 60% in the visible region, the solar cell shows a high energy conversion efficiency of 3.16%, indicating that these three materials of ZnO, CdSe, and Cu_2_O are suitable for application in ETA solar cells. We believe that conversion efficiencies above 5% can be reached by depositing antireflection coating layers onto the back of the glass substrate and reduce the length of the ZnO nanostructures by shortening the growth time, so this kind of ETA solar cell provides a new cheaper alternative to the existing solar cells.

## Competing interests

The authors declare that they have no competing interests.

## Authors' contributions

XHW carried out the experiments and measurements and drafted the manuscript. RBL and DHF participated in the discussion. All authors read and approved the final manuscript.
